# Seasonal temperature variability observed at abyssal depths in the Arabian Sea

**DOI:** 10.1038/s41598-022-19869-z

**Published:** 2022-09-22

**Authors:** M. V. Martin, R. Venkatesan, Robert A. Weller, Amit Tandon, K. Jossia Joseph

**Affiliations:** 1 National Institute of Ocean Technology, Ministry of Earth Sciences, Chennai, India; 2 Present Address: University of Massachusetts Dartmouth, North Dartmouth, MA USA; 3 Woods Hole Oceanographic Institution, Woods Hole, MA USA

**Keywords:** Physical oceanography, Climate sciences, Ocean sciences

## Abstract

The abyssal ocean is generally considered an aseasonal environment decoupled from the variabilities observed at and just below the ocean's surface. Herein, we describe the first in-situ timeseries record of seasonal warming and cooling in the Arabian Sea at a depth of 4000 m. The seasonal cycle was observed over the nearly four-year-long record (from November 2018 to March 2022). The abyssal seasonal temperature cycle also exhibited noticeable interannual variability. We investigate whether or not surface processes influence the near-seabed temperature through deep meridional overturning circulation modulated by the Indian monsoon or by Rossby wave propagation. We also consider if bottom water circulation variability and discharge of the dense Persian Gulf and Red Sea Water may contribute to the observed seasonality.

## Introduction

The physical environment of the deep ocean is characterized by high pressure, low temperature (about two degrees Celsius), sluggish bottom currents, and very low annual organic matter input^1^. Studies have revealed that seawater in the deep basins of the global ocean is warming by 0.1 °C per decade, with higher warming rates in the Arctic and the Southern Ocean^2,3^. Sustained ocean timeseries are critical for characterizing changes in the ocean environment and understanding related marine ecosystem shifts. A significant fraction of oceanographic measurements, especially the timeseries measurements, have been limited to the top few hundred meters. Profiling floats have added the capability to observe the warming of the upper ocean to 2000 m. However, the deep ocean below 2000 m, the largest habitat by volume and area, remains poorly sampled.

The abyssal ocean is generally considered an aseasonal environment. Decade-long ARGO observation suggests that seasonal temperature variations in the ocean decrease with depth and have an amplitude of less than 0.1 °C at ~ 2000 m depth^4^. Nevertheless, current systems^5,6^ and biological fluxes^7,8^ modulate seasonal variations in the abyssal ocean. Recent studies suggest a warming tendency in the abyssal ocean^9^, with several ecosystem implications^10^. The deep ocean warming poses a significant threat to the deep-sea species exerting stress or causing shifts in depth or latitudinal distributions and altering species interactions^11^. The colocation of regions having major shifts in deep-sea ecosystems with the areas having significant changes in surface circulation, temperature and productivity^12^ indicate that the surface ocean variability affects the benthic ecosystems. In the subsequent sections of the paper, we describe the first observational evidence of the seasonal warming and cooling cycle in the abyssal seafloor of the northern Indian Ocean.

## Results

**Figure 1:**
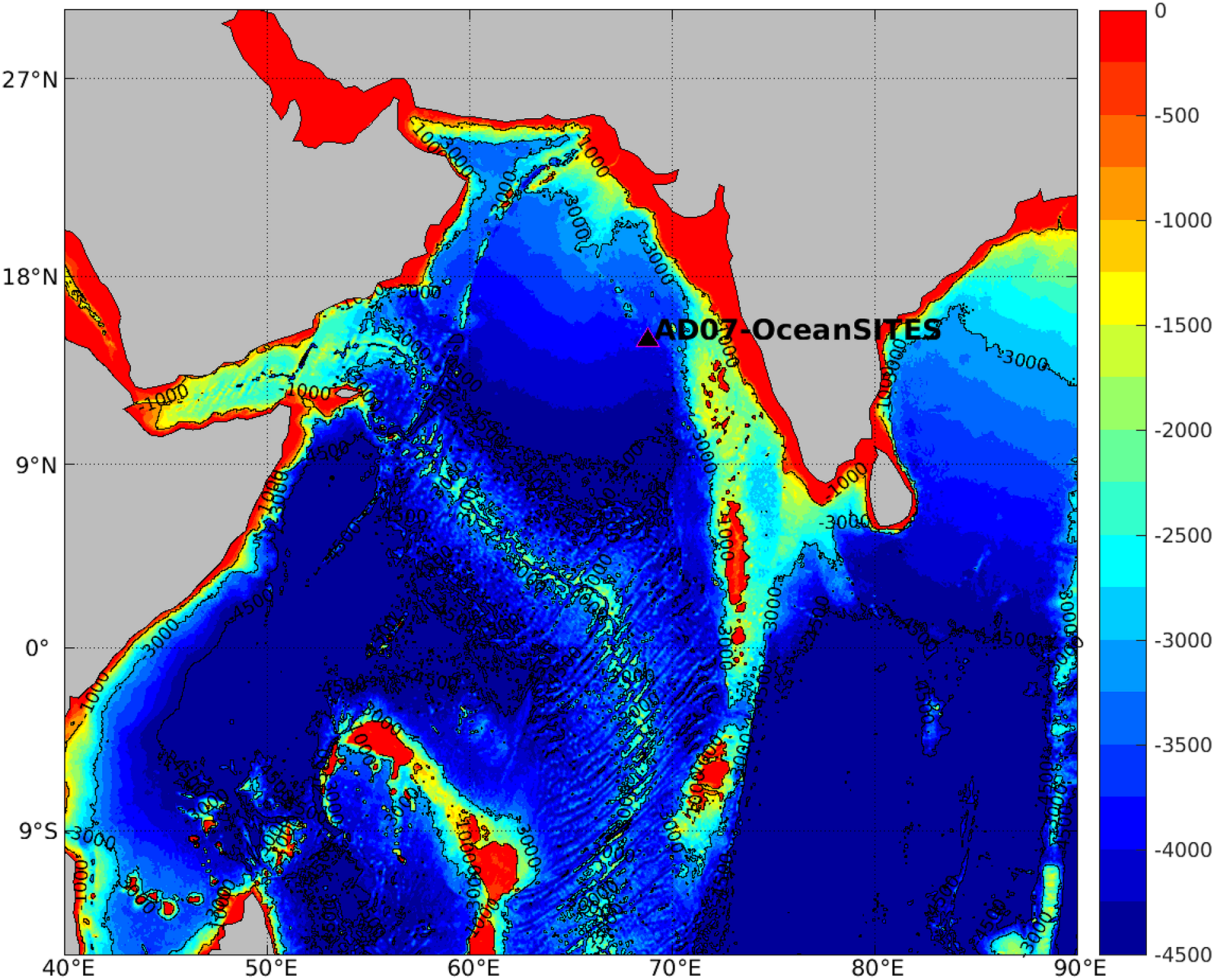
Depth of seabed (in meters) in the Arabian Sea and the surrounding oceanic regions based on ETOPO1 data.

Moored timeseries of near-seafloor temperature and salinity at a depth of ~ 4000 m were obtained in the east-central Arabian Sea location 15° N, 68.8° E (Fig. 1) as part of the OceanSITES program (http://www.oceansites.org). The nearly four-year-long timeseries record (from November 2018 to March 2022) revealed a seasonal temperature cycle in the east-central Arabian Sea mooring location at abyssal depths. The materials and methods section discusses the details of the mooring, sensors and the scope of measurement errors. The salinity data for the first two deployments were not reliable due to the compression of conductivity cell^13^. Additionally, sediment contamination may also have affected the conductivity cell due to the proximity of the sensor to the seabed (~ 10 m) in the initial two deployments. Nevertheless, the temperature sensors returned excellent data, and readings exhibited good continuity between different deployments. The SBE-37 sensor, with a temperature measurement accuracy of 0.002 °C and resolution of 0.0001 °C, recorded the near-seabed in-situ temperature variability bound within 1.715–1.735 °C (Supplementary Fig. S1).

**Figure 2:**
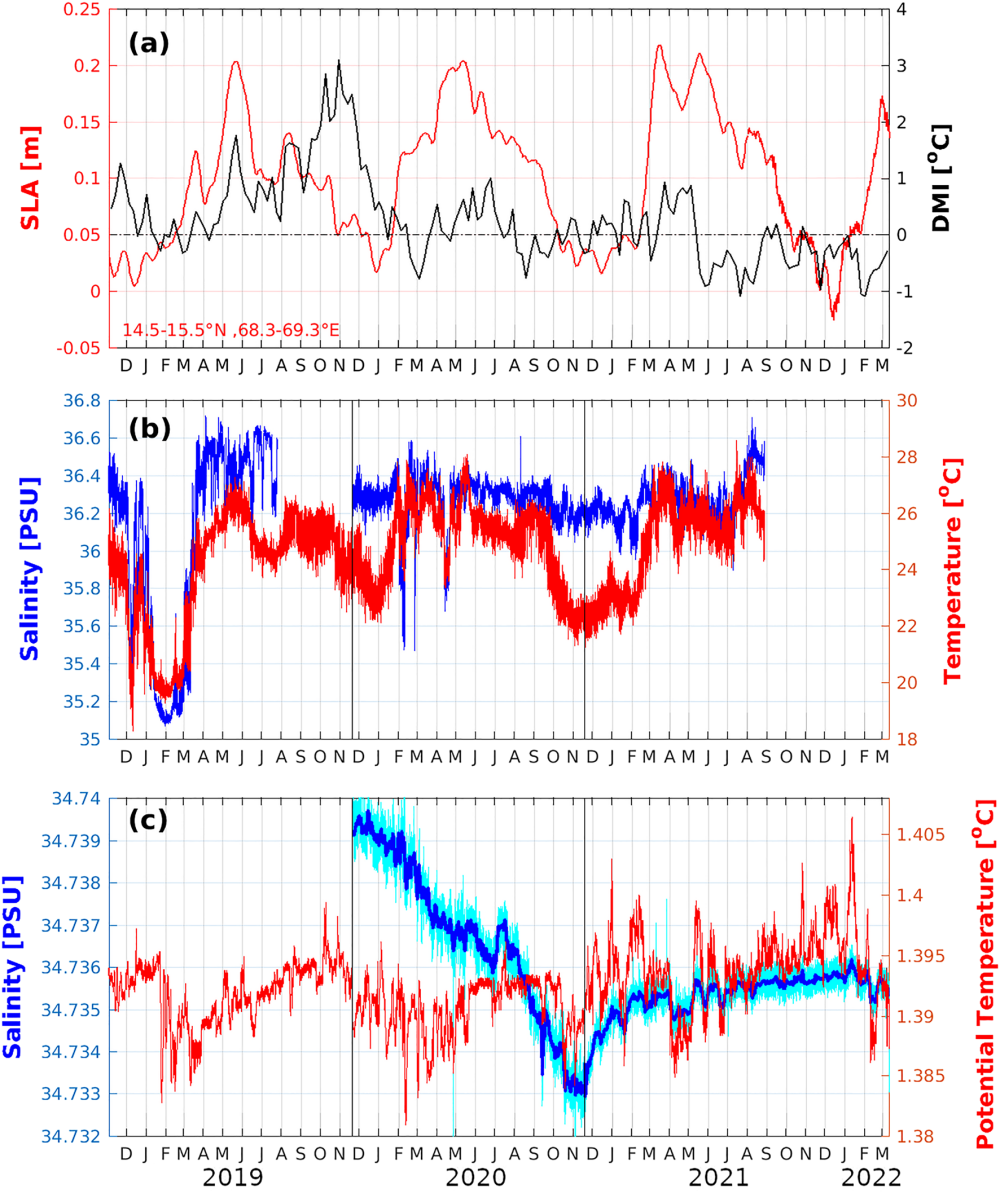
Time series from November 2018 to March 2022 showing (**a**) sea level anomaly in meters averaged in the area bounded by latitudes 14.5–15.5° N and longitudes 68.3–69.3° E (red) and Dipole Mode Index (black). (**b**) Moored buoy observation at 15° N, 68.8° E depicting average temperature in °C (red) and salinity in PSU (blue) in depth from 75 to 100 m. (**c**) Near-seabed time-series depicting15-minute interval potential temperature adjusted to 4500 dBar (θ_4500_), in °C (red) and 3-day running mean of salinity in PSU (blue) while the 15-min interval salinity data is given by cyan line. Black vertical lines in subplots (**b**) and (**c**) indicate the time of redeployment of mooring.

The near-seafloor potential temperature adjusted to 4500 dBar (${\theta }_{4500}$), and salinity are shown in Fig. 2c. The ${\theta }_{4500}$ timeseries revealed seasonal scale cooling events from January to March 2019, from October 2019 to February 2020, and from January to March 2022. Warming events lasting about seven months usually marked the periods between the cooling events. There was considerable interannual variability in the seasonal cycle. The warming phase in 2021–2022 lasted longer than the previous years and cumulated in a maximum recorded ${\theta }_{4500}$ in excess of 1.405 °C. The cooling events in 2018–2019 and 2021–2022 were more abrupt than during 2019–2020. In general, the abyssal temperature variability in the east-central Arabian Sea mooring location was characterized by a warming tendency starting in the spring intermonsoon period and peaking during the winter, followed by a sudden cooling (Fig. 2c). The cooling event in 2020–2021 was the only exception to the pattern as a short warming phase from October 2020 to February 2021 interrupted the seasonal cooling event. Remarkably, the cooling events in the abyssal record roughly coincided with shoaling of seasonal thermocline as evident from cooling and freshening of depth-averaged temperature and salinity respectively in 75–100 m depth (Fig. 2b). However, the variability of temperatures in the seasonal thermocline and the abyssal depths are strictly not correlated during the four-year-long record. In the subsequent section, we propose possible mechanisms that can control the seasonal variability of abyssal temperature and examine the merit of each of them based on the available data.

### Watermass circulation and potential influence of outflow from the marginal seas

The near-seabed water in the Arabian Sea mooring location is believed to originate in the Atlantic and Pacific Oceans. The deeper and bottom waters of the Arabian Sea are composed of the denser Modified North Atlantic Deep Water (MNADW) and the Antarctic Bottom Water (AABW)^14^. Moored timeseries observation in the Atlantic Ocean indicated a low-frequency annual cycle for the AABW^15^. The higher salinity of bottom water in the Arabian Sea than that of the southern sources suggests that the local sources also might influence the bottom watermass. The Persian Gulf and the Red Sea are evaporative basins that discharge high-saline water into the Arabian Sea. The outflow transport from both the Persian Gulf and the Red Sea exhibits a seasonality with a peak discharge into the Arabian Sea during the boreal winter^16,17^. The Persian Gulf Water (PGW) and the Red Sea Water (RSW) in the Arabian Sea are found respectively in 200–400 m, and 500–800 m depths^18^ and their influence on abyssal watermass characteristics is not documented.

It should be noted that the warming (cooling) tendency in the abyssal temperature record is often accompanied by an increase (decrease) in salinity (Supplementary Fig. S1). The compensating changes in temperature and salinity in the abyssal record maintain the *in-situ* density of water nearly unchanged during such events. The simultaneous changes observed in temperature and salinity could be mediated by changes in watermass influx into the region or vertical displacements of isotherms and isohalines. Concurrent measurement of other tracer datasets or currents could provide more insights into the influence of watermass changes on the observed seasonality.

### Rossby wave propagation

The Rossby wave propagation can elevate or depress the water column resulting in temperature variations along different depths. Based on Gravity Recovery and Climate Experiment data, the annual cycle of bottom pressure in the southern tropical Indian Ocean revealed that the Rossby waves have a seafloor signature^19^. Mooring observations in the Pacific Ocean recorded the current and isotherm variability influenced by the propagation of topographic Rossby waves^20^. Previous studies have recorded first- and second-mode annual Rossby waves in the Arabian Sea^21,22^. The Rossby waves propagating in the Arabian Sea are primarily driven by the wind forcing in the equatorial Indian Ocean^23,24^. In-situ observations based on profiling floats in the Arabian Sea suggest that Rossby waves introduce an annual variability below the pycnocline in the Arabian Sea^25^. Hence, we examined whether the simultaneous changes in the temperature and salinity (Supplementary Fig. S1) are caused by the vertical displacements of isotherms and isohalines during the Rossby wave propagation.

**Figure 3:**
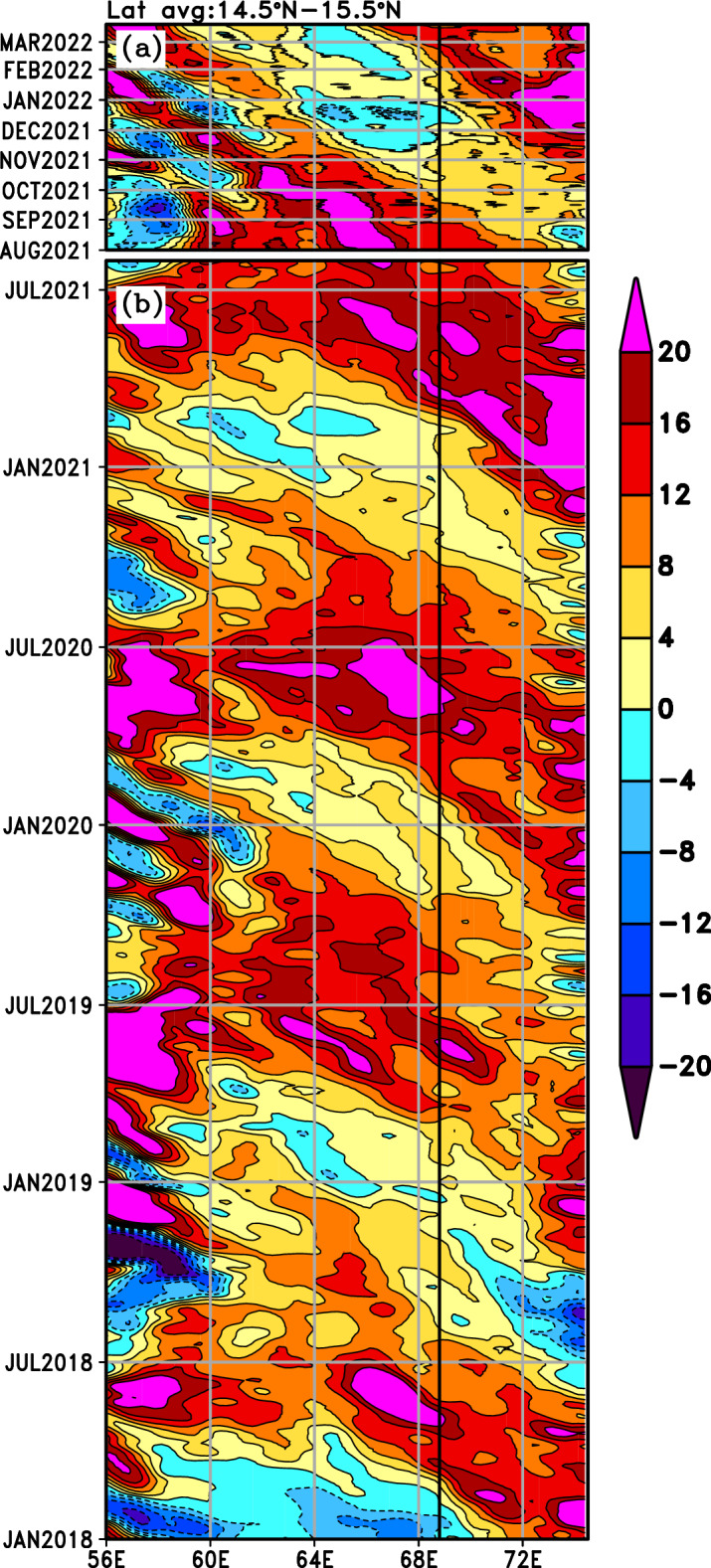
Hovemoller diagram depicting SLA averaged in latitudes from 14.5° N to 15.5° N in the Arabian Sea (**a**) Near Real-Time SLA from August 2021 to March 2022 and (**b**) Delayed Time SLA from January 2018 to July 2021. Black vertical lines in the figure indicate the longitude of the moored buoy.

Hovemoller diagram depicting the temporal evolution of Sea Level Anomaly (SLA) averaged between latitudes from 14.5° N to 15.5° N in the Arabian Sea (Fig. 3) suggests the alternating westward propagation of upwelling and downwelling Rossby waves. The upwelling Rossby waves during the winter seasons 2018–2019 and 2021–2022 were more prominent than the other two events (Fig. 3). Area averaged SLA in a 1° × 1° box centred around the mooring location is shown in Fig. 2a. The area-average timeseries at the mooring location indicate an annual cycle with a low during winter and a high in May prior to onset of monsoon. The SLA variability in the mooring location agrees with the temperature variability in the seasonal thermocline (Fig. 2a,b). However, abyssal temperature variability (Fig. 2c) appears to be decoupled from the SLA variability (Fig. 2a). Thus, the Rossby wave signatures observed in the SLA and seasonal thermocline could not be identified in the abyssal temperature data.

### Deep meridional overturning circulation

The Deep Meridional Overturning Circulation (DMOC) is a meridional circulation that exchanges surface water with deep ocean water^26–28^. The DMOC, with a vertical extent deeper than 3100 m, has seasonal and interannual variability dictated by the surface processes^29,30^. The DMOC in the Indian Ocean is characterized by two counterclockwise cells located south of 30° S and around 10° S, respectively. Wang et al.^31^ decomposed the DMOC into Ekman, thermal wind (geostrophic), external mode, and residual term. The northern cell exhibits monsoon-driven seasonal variability with dominant Ekman mode except for March–April and September–October transition periods^29,31^. The DMOC also highly correlates with the Indian Ocean Dipole (IOD)^29^. The IOD events are commonly identified based on a dipole mode index (DMI)^32^, which is calculated as the differences in SST anomalies averaged in two boxes located in the western (10° S–10° N, 50–70° E) and southeastern (10° S–0° N, 90–110° E) tropical Indian Ocean regions. Weekly mean DMI from January 2018 to December 2021 (Fig. 2a) indicates the occurrence of the positive phase of IOD during 2019. However, no significant variability associated with the IOD was observed in the abyssal timeseries data (Fig. 2c). Potential contribution of DMOC on seasonal abysal temperature variability has to be further investigated as the Arabian Sea is landlocked on all three sides except for the south and forced predominantly by the seasonally reversing monsoonal winds.

## Discussion

Moored timeseries measurements in the east-central Arabian Sea from November 2018 to March 2022 revealed a pronounced seasonal signal in the abyssal temperature. In general, the abyssal temperature variability in the region is characterized by a warming tendency starting during the spring intermonsoon period and peaking during the winter, followed by sudden cooling. We have further examined the plausible mechanisms that could govern the warming and cooling tendencies in the abyssal Ocean. The analysis revealed concurrent changes in salinity and temperature during such warming and cooling events, pointing to the potential influences of changes in watermass or flow rate as recorded in the Atlantic by an array of moored buoy networks^15,33^. Previous studies from the Pacific Ocean identified modulations in temperature and current at a depth of 4500 m under the influence of Rossby wave propagation. However, an examination of SLA suggested that the Rossby wave propagation might not have a significant influence on the observed abyssal temperature variability in the east-central Arabian Sea location. The DMOC influenced by monsoon wind forcing and vertical extent deeper than 3100 m might have an impact on the near-seafloor temperature variability. Further, studies are required to identify the processes that govern the seasonal temperature variability in the east-central Arabian Sea.

The seasonal signal in the abyssal temperature could also impact the benthic ecosystems^34^ the context of climate change. The seasonal temperature variability observed in the abyssal depths of the Arabian Sea also assumes significance in the context of studies using repeat hydrography measurements to decipher warming signals in the abyssal waters. The abyssal warming trends observed in studies based on the repeat hydrography data^2,34,35^ are about 0.005 °C/year, which is smaller than the seasonal temperature variability observed in the Arabian Sea. Repeat hydrography done every 5 or 10 years cannot capture the temporal variability that a deep-moored sensor does. Besides, such studies might alias the seasonal cycle and incorrectly ascribe change over the years to low-frequency climate change. Timeseries record of deep temperature and salinity are also useful to assess and refine deep mixing parameterizations in climate models.

## Materials and methods

Near-seabed timeseries data from the east-central Arabian Sea location, 15° N, 68.8° E, were gathered by Ocean Moored buoy for North Indian Ocean (OMNI) mooring AD07^36^. The mooring was equipped with three additional SeaBird SBE-37-IM sensors in the abyssal water column from November 2018 to March 2022 as part of the OceanSITES program. This study primarily focuses on timeseries near-seabed temperature and salinity data from ~ 4000 m. The mooring was serviced three times during the period and the 7000 m rated SBE-37-IM sensors were replaced by calibrated sensors during each servicing. The temperature (conductivity) data of SBE-37 in the range − 5° to 45 °C (0–7 S/m) has an initial accuracy of ± 0.002 °C (± 0.0003 S/m) and stability of 0.0002 °C/month (0.0003 S/m/month). At the same time, the pressure data has an initial accuracy of ± 0.1% range and stability of 0.05% range/year. SBE-37 s sensors were reported to have a sensitivity to pressure change due to compression of the sensor^13^, which caused the pressure and conductivity data to increase rapidly for ~ 50 days before stabilizing. The near-seabed deployments of SBE-37 sensors in the Arabian Sea mooring also showed a similar pattern, which caused a rapid artificial increase in salinity data during the initial few days of the deployment (Supplementary Fig. S1). The first two deployments of near-seabed CT sensors (November 2018 to November 2020) were 10 m above the seabed. The proximity to the seabed could have led to the conductivity cell ingesting bottom sediment at the time of deployment or from the bottom nepheloid layer^13^, leading to erroneous conductivity and salinity data (Fig. 2). All subsurface sensors, especially the near-surface sensors, are prone to biofouling^37^, which could also introduce drifts in data. Post-retrieval tests were done for SBE-37 sensors immediately after retrieval following Navaneeth et al.^38^, and the near-seabed sensors for all three deployments showed no noticeable drifts in temperature and conductivity. It is possible that the fast ascending phase during the retrieval procedure flushed conductivity cells and improved data quality during the post-retrieval tests. In the subsequent deployment (November 2020), the sensor was placed 50 m above the seafloor, leading to a significant improvement in salinity data.

The OMNI buoys use a slack mooring configuration. Consequently, the near-seabed sensor exhibited depth variation of up to 6 m, most pronounced during monsoon season (Supplementary Fig. S1). A mooring failure in August 2021 detached the subsurface oceanographic sensors from the surface buoy. Even though the depth variations of the near-seabed sensor were restricted following the mooring failure in August 2021, the temperature and salinity timeseries did not exhibit any noticeable changes (Supplementary Fig. S1). Moreover, the temperature timeseries showed excellent continuity between different deployments despite a change of deployment depth by 40 m of the near-seabed sensor for the third deployment (Fig. 2). The lapse rate of temperature and salinity at these depths are minimal to introduce any notable noise in the data associated with depth variability during the entire timeseries record.

Potential temperature adjusted to 4500 dBar (${\theta }_{4500}$) was calculated following the Thermodynamic Equation of SeaWater 2010 (TEOS-10). We have used mean salinity during the third deployment (November 2020 to March 2022) and mean pressure from individual deployments for the ${\theta }_{4500}$ calculation. Our analysis (Supplementary Fig. S2) revealed that as the relative changes in salinity and pressure throughout the record were very small, replacing instantaneous measurements of pressure and salinity in the calculation with the mean of data for the entire record does not introduce any noticeable deviation in ${\theta }_{4500}$. The gridded daily global estimates of near-real-time and delayed time SLA based on Ssalto/Duacs altimeter products produced and distributed by Copernicus Marine and Environment Monitoring Service (CMEMS) are used to examine Rossby wave signal. The weekly mean Dipole Mode Index data used in this study are obtained from Ocean Observation Panel for Climate (https://stateoftheocean.osmc.noaa.gov/).

## Supplementary Information

Supplementary Figures.

## Data Availability

Moored buoy datasets used herein are available at https://incois.gov.in/portal/datainfo/buoys.jsp. The SLA data are obtained from https://marine.copernicus.eu/ Weekly mean dipole mode index data is obtained from https://stateoftheocean.osmc.noaa.gov/.
