# Supplementary material for: Seasonal temperature variability observed at abyssal depths in the Arabian Sea
Source: Sci Rep. 2022 Sep 22;12:15820. doi: 10.1038/s41598-022-19869-z (PMC9500021; doi:10.1038/s41598-022-19869-z)
Supplement: Supplementary file 1 — Supplementary Figures. [file 41598_2022_19869_MOESM1_ESM.pdf]

Seasonal temperature variability observed at abyssal depths in the Arabian Sea

M. V. Martin[1*], R. Venkatesan[1], Robert A. Weller[2], Amit Tandon[3] and K. Jossia Joseph[1]
[1] National Institute of Ocean Technology, Ministry of Earth Sciences, Chennai, India
[2] Woods Hole Oceanographic Institution, Woods Hole, Massachusetts, USA
[3] University of Massachusetts Dartmouth, North Dartmouth, Massachusetts, USA
* Corresponding author: vmartinmathew@gmail.com

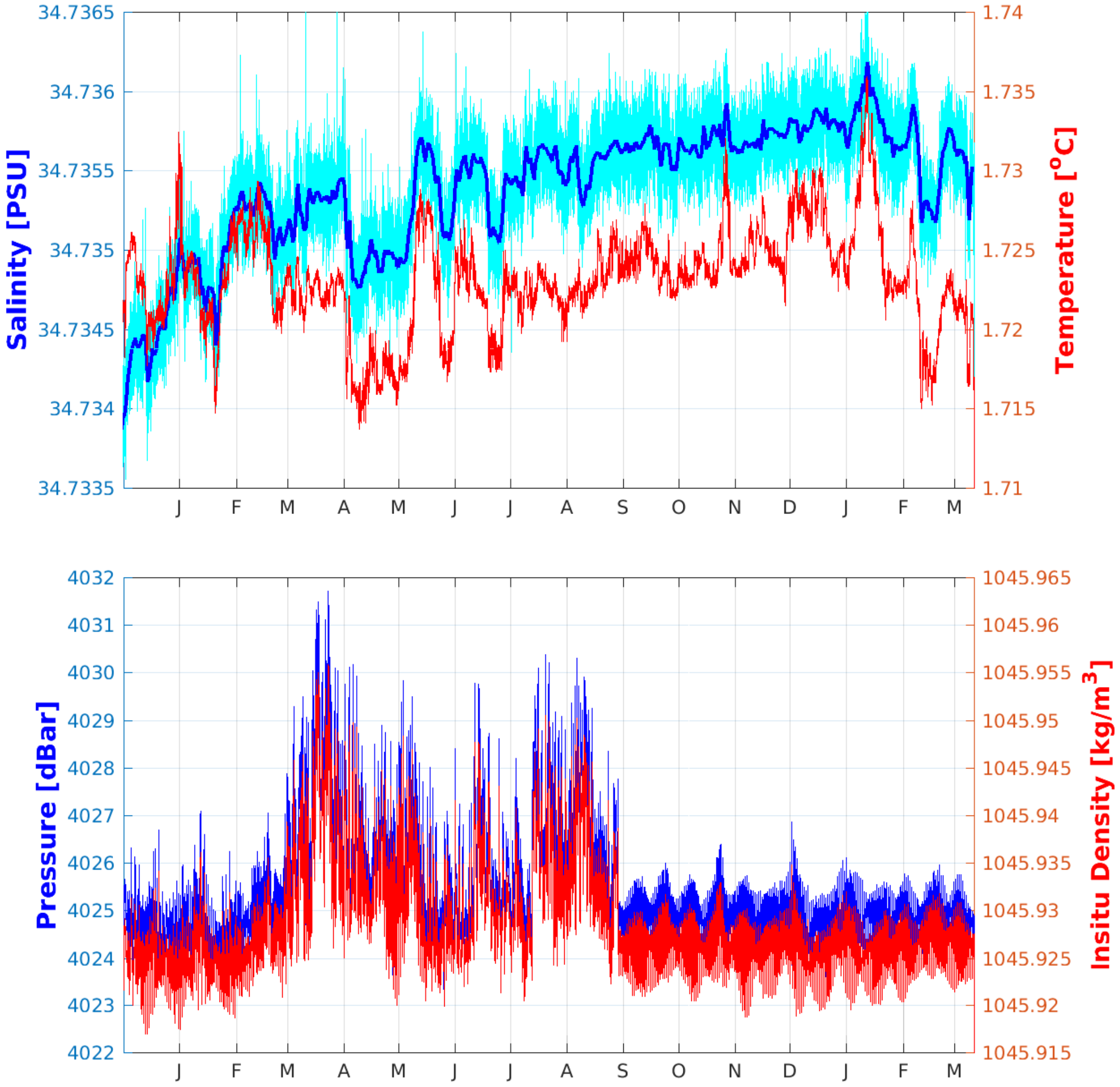

Supplementary Figure 01: Timeseries record from December 2020 to March 2022 based on moored buoy observation at 15°N,68.8°E depicting (a) near-seabed moored time-series measurement of salinity in PSU (blue) and temperature in degree Celsius (red) (b) Pressure measured by the SBE-37 sensor. The surface buoy was cut-off from the mooring in August 2021.

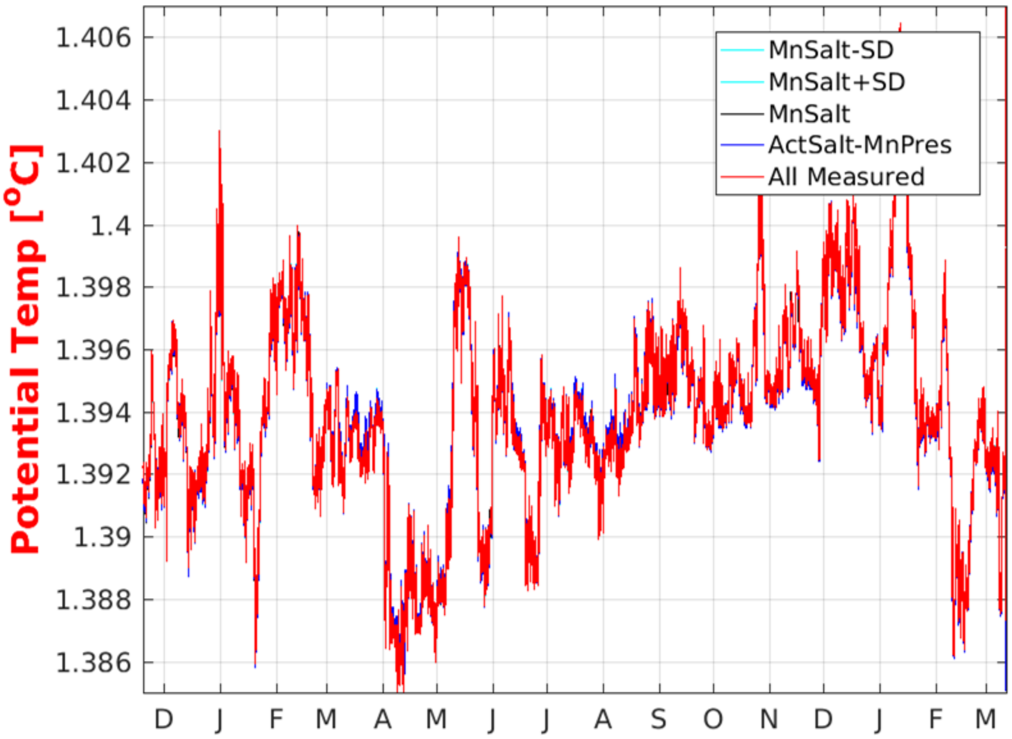

Supplementary Figure 02: Potential temperature adjusted to 4500 dBar (θ_{4500}) based on near-seabed CT sensor data from OceanSITES from November 2020 to March 2022. The calculations using mean salinity and mean pressure (black line) during the deployment did not deviate significantly with respect to the θ_{4500} obtained from instantaneous salinity and pressure (red line) data.
